# Zn(II)-to-Cu(II) Transmetalation in an Amide Functionalized Complex and Catalytic Applications in Styrene Oxidation and Nitroaldol Coupling

**DOI:** 10.3390/molecules25112644

**Published:** 2020-06-06

**Authors:** Anup Paul, Luísa M. D. R. S. Martins, Anirban Karmakar, Maxim L. Kuznetsov, M. Fátima C. Guedes da Silva, Armando J. L. Pombeiro

**Affiliations:** Centro de Química Estrutural, Instituto Superior Técnico, Universidade de Lisboa, 1049-001 Lisboa, Portugal; anirban.karmakar@tecnico.ulisboa.pt (A.K.); max@mail.ist.utl.pt (M.L.K.)

**Keywords:** amide functionalized, transmetalation, DFT calculations, oxidation of styrene, nitroaldol

## Abstract

The mononuclear zinc(II) complex *cis*-[ZnL_2_(H_2_O)_2_] (**1**; L = 4-(pyridin-3-ylcarbamoyl)benzoate) was synthesized and characterized. By soaking crystals of **1** in a mixture of DMF-H_2_O solution containing a slight excess of Cu(NO_3_)_2_ × 3H_2_O a transmetalation reaction occurred affording the related copper(II) complex *trans*-[CuL_2_(H_2_O)_2_] (**2**). The structures of the compounds were authenticated by single crystal X-ray diffraction revealing, apart from a change in the isomerism, an alteration in the relative orientation of the chelating carboxylate groups and of the pyridine moieties. H-bond interactions stabilize both geometries and expand them into two-dimensional (2D) networks. The transmetalation was confirmed by SEM–EDS analysis. Moreover, the thermodynamic feasibility of the transmetalation is demonstrated by density-functional theory (DFT) studies. The catalytic activities of **1** and **2** for the oxidation of styrene and for the nitroaldol (Henry) C-C coupling reaction were investigated. The copper(II) compound **2** acts as heterogeneous catalyst for the microwave-assisted oxidation of styrene with aqueous hydrogen peroxide, yielding selectively (>99%) benzaldehyde up to 66% of conversion and with a turnover frequency (TOF) of 132 h^−1^. The zinc(II) complex **1** is the most active catalyst (up to 87% yield) towards the nitroaldol (Henry) coupling reaction between benzaldehyde and nitro-methane or -ethane to afford the corresponding β-nitro alcohols. The reaction of benzaldehyde with nitroethane in the presence of **1** produced 2-nitro-1-phenylpropanol in the *syn* and the *anti* diastereoisomeric forms, with a considerable higher selectivity towards the former (66:34).

## 1. Introduction

Transmetalation is commonly associated with an organometallic compound and involves a transfer of organic groups or ligands from one metal to another [1,2,3]. It thus provides a common and convenient method for preparing various organo-transition metal complexes, which are otherwise difficult to synthesize via direct metal-ligand self-assembly. Such phenomenon is well documented and has expanded to other types of materials [4,5,6,7,8,9,10,11]. The process usually proceeds through either ‘‘direct exchange’’, where the incoming metal does not alter the topology of the frameworks or through ‘‘disruptive exchange’’ in which the geometry of the transmetalation complex differs from the original structure as a result of the metal exchange.

Several metal-organic frameworks (MOFs) have been employed in transmetalation to synthesize novel materials which would otherwise be difficult to obtain [12,13,14,15]. The role of ligands in the process cannot be ruled out.

Previously, the presence of a carboxylate and a pyridyl moieties in the ligand framework of (4-(pyridin-3-ylcarbamoyl)benzoate) was used for the synthesis of MOFs or coordination polymers (CPs) by Luo et al. [16]. The presence of a carboxylate and pyridyl moieties was used for the synthesis of MOFs or coordination polymers (CPs) but, in the present study, we failed to generate such types of compounds through hydro/solvothermal reaction using a zinc salt. Instead, unexpectedly a mononuclear molecular zinc(II) complex was obtained, being susceptible to undergo transmetalation with copper(II) (see below).

In pursuing our interest on the synthesis of metal-based complexes for application in catalysis, we have been using amide functionalized ligands primarily because the amide backbone in the ligand framework can provide a basic environment and facilitate the catalytic reaction [17,18,19,20]. Furthermore, the presence of multiple coordinating sites in the ligand skeleton can provide favorable stretched frameworks having a large surface area, also advantageous for catalysis.

Concerning a catalytic application, the preparation of benzaldehyde is an important process as this compound is a valuable chemical for perfumery, dyestuff, pharmaceutical and agro-chemical industries [21,22,23,24,25]. Although it can be obtained by oxidation of benzyl alcohol, traditionally benzaldehyde is produced by catalytic partial oxidation of toluene or by hydrolysis of benzylidene chloride [26,27]. Both processes present low yields, harsh conditions and a significant generation of waste. Therefore, there is a need to find more environmentally friendly processes using green oxidation agents (e.g., hydrogen peroxide) and heterogeneous catalyst systems.

On the other hand, the nitroaldol (Henry) reaction is also relevant, being widely used to build C–C bonds by the formation of β-nitro alcohols (from the Csp^3^–Csp^2^ coupling reaction between a nitroalkane and a carbonyl compound), which are important synthetic precursors. This reaction can be carried out with homogeneous and heterogeneous catalysts, as well as with alkali metal hydroxides, alkoxides or amines [28,29,30,31]. We have already reported a variety of homogeneous and heterogeneous catalysts for this kind of reaction [17,18,32,33,34,35]. Despite several progresses, good enantio- and diastereoselectivity is rarely achieved. Thus, also for this reaction, a need for improved catalytic systems remains.

Herein, we present the synthesis and structural analysis of the new mononuclear complex of zinc(II) *cis*-[ZnL_2_(H_2_O)_2_] (**1**, L = 4-(pyridin-3-ylcarbamoyl)benzoate) and of the derived copper(II) compound *trans*-[CuL_2_(H_2_O)_2_] (**2**). **1** was synthesized by hydrothermal reaction, whereas its post-synthetic modification led to the formation of compound **2** through transmetalation, which is irreversible. Complexes **1** and **2** are shown to catalyze the oxidation of styrene towards benzaldehyde and the Henry reaction of an aldehyde with a nitro alkane to form a β-nitro alcohol. The mononuclear Cu(II) complex **2** acts as a heterogeneous catalyst for the microwave-assisted selective oxidation of styrene to benzaldehyde, whereas the Zn(II) complex **1** converts successfully benzaldehyde into β-nitro alcohols.

## 2. Experimental Section

### Materials and Methods

Solvents were dried and distilled before their use. Reagents were obtained from commercial sources and used without further purification. Methyl-4-(chlorocarbonyl)benzoate was prepared following a procedure reported earlier [36]. FT-IR spectra were recorded on a Bruker Vertex 70 (Bruker Corporation, Ettlingen, Germany) instrument in KBr pellets. ^1^H (300 MHz) and ^13^C (75.45 MHz) NMR spectra were obtained at room temperature (RT) on a Bruker Avance II + 300 (UltraShield™Magnet, Rheinstetten, Germany) spectrometer using tetramethylsilane [Si(CH_3_)_4_] as an internal reference. Carbon, hydrogen, and nitrogen elemental analyses were carried out by the Microanalytical Service of the Instituto Superior Técnico, Lisboa, Portugal. Powder X-ray diffraction (PXRD) was conducted in a D8 Advance Bruker AXS (Bragg Brentano geometry, Bruker, Madison, WI, USA) theta-2-theta diffractometer, with copper radiation (Cu Kα, λ = 1.5406 Å) and a secondary monochromator, operated at 40 kV and 40 mA. Flat plate configuration was used, and the typical data collection range was between 5° and 40°. Thermal properties were performed using a PerkinElmer Instrument system (STA6000, Perkin-Elmer, Boston, MA, USA) at a heating rate of 10 °C min^−1^ under a dinitrogen atmosphere flow rate of 30 mL/min. The synthesized compounds were characterized in terms of structural and optical properties by scanning electron microscopy (SEM) and energy-dispersive X-ray spectroscopy (EDX) using a scanning electron microscope JEOL 7001 F (JEOL, Akishima, Tokyo, Japan) with Oxford light elements EDX detector.

## 3. Synthesis and Characterization

### 3.1. Synthesis of 4-(Pyridin-3-Ylcarbamoyl)Benzoic Acid (HL)

The pro-ligand was synthesized according to a reported procedure [16,36].

### 3.2. Synthesis of cis-[ZnL_2_(H_2_O)_2_] *(**1**)*

Zn(NO_3_)_2_·6H_2_O (40 mg; 0.134 mmol) dissolved in 1 mL of water was added to a 1 mL DMF solution of **HL** (33 mg; 0.135 mmol). The mixture was sealed in an 8 mL glass vessel and heated at 75 °C for 24 h. Upon standing at room temperature for two days, crystals were formed, isolated by filtration, washed with DMF followed by deionized water, and dried in air. Yield: 70% (based on Zn). Anal. Calcd for C_26_H_22_ZnN_4_O_8_: C, 53.49; H, 3.80; N, 9.60. Found: C, 53.53; H, 3.83; N, 9.62. IR (KBr, cm^−1^): 3320 (bs), 1648 (s), 1593 (m), 1584 (s), 1482 (s), 1408 (m), 1385 (m), 1295 (m), 1234 (m), 1018 (m), 901 (m), 865 (w), 841 (w), 799 (w), 701 (w).

### 3.3. Synthesis of trans-[CuL_2_(H_2_O)_2_] *(**2**)*

An aqueous solution of Cu(NO_3_)_2_ × 3H_2_O (25 mg; 0.103 mmol) was added to the synthesized complex **1** (50 mg; 0.085 mmol) soaked in DMF:H_2_O (1:1, *v*/*v*) and left undisturbed at room temperature. Deep blue crystals formed after two days, were collected by filtration, washed with DMF, deionized water, methanol, and dried in air. Yield: 60% (based on Cu). Anal. Calcd for C_26_H_22_CuN_4_O_8_: C, 53.49; H, 3.80; N, 9.60. Found: C, 53.50; H, 3.85; N, 9.62. IR (KBr, cm^−1^): 3288 (bs), 1653 (s), 1631 (w), 1593 (w), 1530 (s), 1483 (w), 1386 (m), 1375 (w), 1294 (m), 1335(w), 1105 (w), 1020 (w), 799 (w), 736 (m), 665 (m).

## 4. Crystal Structure Determination

Single crystals of **1** and **2** were leapt in cryo-oil, mounted in a nylon loop and measured at room temperature. Intensity data were collected using a Bruker APEX-II PHOTON 100 diffractometer (Bruker, Madison, WI, USA) with graphite monochromated Mo-Kα (λ = 0.71069) radiation. Data were collected using phi and omega scans of 0.5° per frame, and a full sphere of data was obtained. Cell parameters were retrieved using Bruker SMART [37] software and refined using Bruker SAINT [37] on all the observed reflections. Absorption corrections were applied using SADABS [38]. Structures were solved by direct methods by using the SIR97 program and refined with SHELX-2014/7 [39,40]. Calculations were performed using the WinGX System–Version 2014.1 [41]. The hydrogen atoms were inserted in calculated positions and included in the refinement using the riding-model approximation; Uiso (H) were defined as 1.2 Ueq of the parent carbon atoms for phenyl residues. The water hydrogen atoms were found in the difference Fourier map and were included in the refinement using the riding-model approximation with the Uiso(H) defined as 1.5 Ueq of the parent O-atom. PLATON/SQUEEZE [42] was used to correct the data. Crystallographic data are summarized in Table 1 and selected hydrogen bonding interactions are presented in Table S1. CCDC 2000481-2000482 contains the supplementary crystallographic data for this paper. These data can be obtained free of charge from The Cambridge Crystallographic Data Centre via www.ccdc.cam.ac.uk/data_request/cif.

## 5. Computational Details

The full geometry optimization of all structures was carried out at the DFT level of theory by using the M06 functional [43] with the help of the Gaussian 09 program package [44]. No symmetry operations were applied. The standard basis set 6-31G* was applied for all atoms. Solvent effects were taken into account in single-point calculations based on gas phase geometry using the “density-based” solvation model (SMD) solvation model with water and DMF selected as solvents [45]. The 6-311+G** and Def2-TZVP basis sets were applied in these calculations for non-metal and metal atoms, respectively. The solvent effect for the 1:1 mixture of water and DMF was approximated as the average value obtained for pure water and DMF solvents. The optimization of complex **1** was additionally performed at the SMD(H_2_O)-B3LYP/6-31+G* and SMD(H_2_O)-ωB97XD/Def2-TZVP levels of theory. The Cartesian atomic coordinates of the calculated equilibrium structures are presented in Table S2.

The Hessian matrix was calculated analytically for the optimized structures to prove the location of correct minima (no imaginary frequencies) and to estimate the thermodynamic parameters, with the latter calculated at 25 °C. The Gibbs free energies in solution, G_s_, discussed in the text were obtained as G_s_ = E_s_(6-311 + G**/Def2-TZVP) – E_g_(6-31G*) + G_g_(6-31G*), where E_s_ and E_g_ are total energies in solution and gas phase and G_g_ is gas phase Gibbs free energy obtained using the indicated basis set.

## 6. Catalytic Studies

Typical oxidation of styrene was performed under microwave irradiation (10 W) in a focused microwave Anton Paar Monowave 300 (Anton Paar GmbH, Graz, Austria) discover reactor fitted with a rotational system and an IR temperature detector. A 10 mL capacity cylindrical Pyrex tube with a 13 mm internal diameter was used: catalyst (10 μmol), the substrate (1.0 mmol), hydrogen peroxide (30% *w*/*w* aq. sol., 2.0 mmol), 1.5 mL of acetonitrile and 100 μL of chlorobenzene as internal standard were stirred at 80 °C for the desired reaction time. During the reaction, small aliquots were taken, cooled down, centrifuged (to separate the catalyst from the sample), and analyzed by GC–MS using the internal standard method for quantification. GC–MS analyses were conducted at a Perkin-Elmer Clarus 600 C (Shelton, CT, USA) apparatus, using He as the carrier gas, with an ionization voltage of 70 eV and an SGE BPX5 column (30 m × 0.25 mm × 0.25 µm). The comparison of the products retention times with those of known reference compounds enabled their identification. Moreover, their mass spectra fragmentation patterns were compared with those obtained from the National Institute of Standards and Technology (NIST) spectral library of the computer software of the spectrometer. The values of conversion considered result from two concordant assays. Blank reactions were performed for the best conditions in the absence of complexes **1** or **2** and confirmed that almost no oxidation occurs. In some runs under the optimized conditions, 2,2,6,6-tetramethylpiperidine-1-oxyl (TEMPO, 1.0 mmol) was added to the initial reaction mixture as a radical scavenger.

The recyclability of complex **2** was examined, by recovering it by centrifugation after the reaction being cooled down, washing with ethanol, and drying overnight. A new cycle was run with the recovered catalyst by addition of new typical portions of all other reagents. After completion of the reaction, the products were analyzed as above-mentioned.

The experiments for the Henry reaction were carried out in a glass reactor. A mixture of the nitroalkane (1.5 mmol), benzaldehyde (0.5 mmol), catalyst **1** (50 μmol) and 3 mL of solvent (deionized water, methanol, dichloromethane, dichloroethane, THF, or acetonitrile) was stirred up to 24 h at room temperature to 60 °C. After the desired reaction time, the solution was extracted with diethyl ether (3 × 5 mL), the combined extracts dried over anhydrous Na_2_SO_4_, and then the mixture was filtered off. The diethyl ether was removed in vacuo and the organic residue was analyzed by ^1^H NMR spectrometry, in CDCl_3_, for quantification of the formed β-nitro alcohols (as no other products were detected). The *anti*:*syn* diastereoisomer ratio of 1-phenyl-2-nitropropanol was determined taking into account the vicinal coupling constants values between the α-O–C–H and the α-N–C–H protons for the isomers: *anti*, *J* = 7–9 Hz; *syn*, *J* = 3.2–4 Hz) [46,47].

## 7. Results and Discussion

### Syntheses and Characterization

**Scheme 1:**
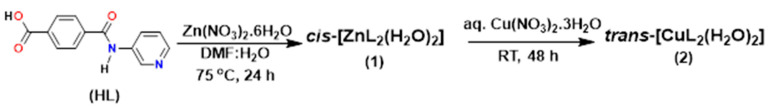
Syntheses of compounds **1** and **2**.

**Figure 1:**
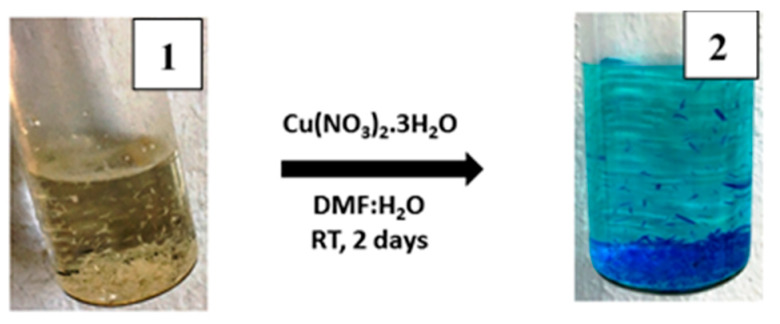
Conversion of **1** to **2**.

The pro-ligand **HL** was prepared according to the reported modified procedure [16]. Compound **1** was synthesized by the hydrothermal reaction between Zn(NO_3_)_2_.6H_2_O and **HL** in a DMF:H_2_O solvent mixture (Scheme 1 and experimental section for synthetic details). The transmetalation reaction to afford **2** was carried out by soaking crystals of **1** in a DMF:H_2_O (1:1, *v*/*v*) solvent mixture containing a slight excess of aqueous Cu(NO_3_)_2_ × 3H_2_O and leaving it undisturbed at room temperature for two days (Figure 1). Both compounds were characterized by elemental analysis, IR, SEM–EDS and single crystal X-ray diffraction analyses. It is worthy to mention here that we failed the attempts to achieve the direct synthesis of the transmetalated Cu(II) complex **2** by deprotonating the pro-ligand **HL** with a base, such as KOH, NaOH or NH_4_OH, and in situ reaction with a copper(II) salt like Cu(NO_3_)_2_ × 3H_2_O or CuCl_2_ × 2H_2_O, at room temperature or heating in methanol or in a DMF:H_2_O mixture.

The FT-IR spectrum of **HL** displays a peak at ca. 1705 cm^−1^ assigned to the asymmetric stretching vibration of the carboxyl group which, upon coordination to the metal cations shifts to the lower frequency values of 1648 cm^−1^ (in **1**) and 1653 cm^−1^ (in **2**) (Figure S1).

## 8. Crystal Structure Analysis

**Figure 2:**
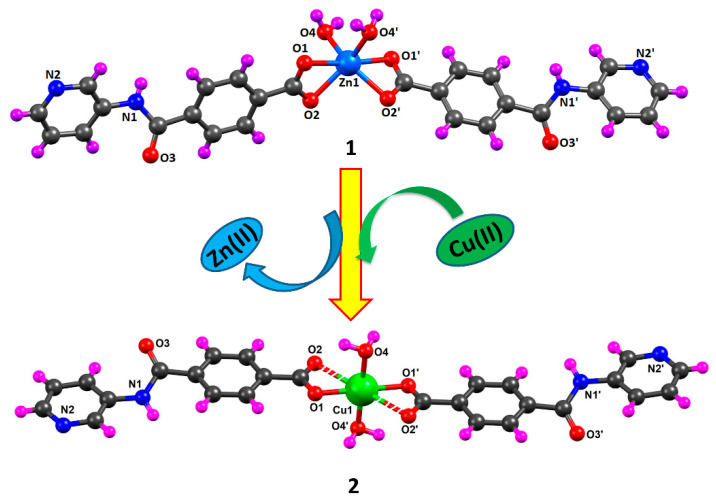
Crystal structures of compounds **1** and **2**.

The crystal structures of **1** and **2** were determined by single-crystal X-ray diffraction analysis and are presented in Figure 2. Crystallographic data of the compounds **1** and **2** are presented in Table 1 and hydrogen bond contacts are detailed in Table S1 (Supplementary Materials).

The asymmetric units of the compounds include one metal ion (Zn^2+^ in **1** or Cu^2+^ in **2**), one deprotonated **L**^−^ ligand binding the cations through chelating (in **1**) or monodentate (in **2**) carboxylate moieties, and one water molecule (Figure 2). Symmetry expansion reveals mononuclear complexes where the water ligands are in mutual *cis* (in **1**) or *trans* (in **2**) position. In view of the constraints derived from the chelating mode of **L**^−^ in **1** the six-coordinate geometry is better described as trigonal prismatic with the angle between the least-square planes of the carboxylate groups being of 78.06°. In **2**, the environment around the metal cation can be envisaged as square planar but the long-range interaction between Cu1 and O2 or O2′-carboxylate atoms (Cu1⋅⋅⋅O2/O2′ 2.606 Å), being shorter than the sum of the van der Waals radii of Cu (1.40 Å) and O (1.52 Å), can be considered as giving rise to a highly distorted octahedral geometry. The Zn-O and Cu-O bond distances are in the range of 2.0197(16)-2.2020(15) Å and 1.955(4)-1.968(3) Å, respectively. The N2 and N2′ atoms are uncoordinated, displaying the *syn* orientation in **1** and the *anti* in **2** (Figure 2).

**Figure 3:**
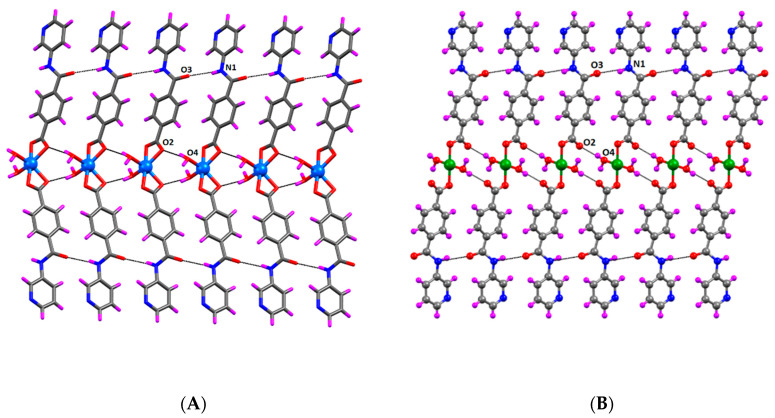
Hydrogen bonded networks of compounds **1**(**A**) and **2**(**B**).

Both compounds are involved in intermolecular H-interaction (Table S1) where the water ligands act as donors to an O2 and the N2 atoms of vicinal complex molecules (donor⋅⋅⋅acceptor distances in the 2.667(6)–2.722(2) Å range; ∠D−H⋅⋅⋅A between 151(6) and 168(2) °); the N1, in turn, donates to the O3 atoms, such contacts being less intense than the previous ones (donor⋅⋅⋅acceptor distances and D−H⋅⋅⋅A angles of 3.006(3) Å and 168(3)° in **1**, and 3.013(6) Å and 159(6)° in **2**). Resulting from such interactions infinite two-dimensional (2D) networks of **1** or **2** are generated (Figure 3) spreading along the (10-1) or the (100) planes, respectively.

## 9. DFT Calculations

**Figure 4:**
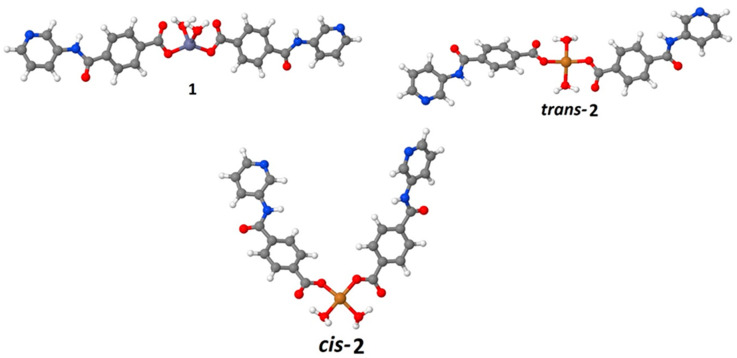
Equilibrium structures of **1**, *trans*-**2** and *cis*-**2**.

In order to understand the thermodynamic feasibility of the conversion of **1** to **2** through transmetalation, DFT calculations were carried out. The DFT calculations with various functionals and basis sets (gas-M06/6-31G*, SMD(H_2_O)-B3LYP/6-31+G*, and SMD(H_2_O)-ωB97XD/def2-TZVP) indicated that in both gas and liquid phases the structure of the Zn complex **1** corresponds to a distorted tetrahedral coordination sphere with both 4-(pyridin-3-ylcarbamoyl)benzoate ligands being monodentate (the observed long range Zn-O interaction of the chelating carboxylate groups is discarded) (Figure 4). Therefore, the structure of complex **1** in the solid state is controlled by crystal packing effects, first of all by multiple intermolecular interactions described above.

In accord with the calculations, the experimentally isolated *trans*-isomer of **2** is more stable than the corresponding *cis*-isomer in all water, DMF, and 1:1 water/DMF mixture by 1.6, 2.1 and 1.8 kcal/mol, respectively. The coordination sphere in both optimized structures *trans*-**2** and *cis*-**2** deviates significantly from the square planar configuration with the *trans*-O–Cu–O angles of 148–158°. The carbonyl groups of the ligands form intramolecular H-bonds with coordinated water molecules.

The calculated Gibbs free energy of reaction (1) in the 1:1 water/DMF mixture is significantly negative (−12.7 kcal/mol) indicating thermodynamic preference of the transmetalation observed experimentally.

**1** + [Cu(H_2_O)_6_]^2+^ → *trans*-**2** + [Zn(H_2_O)_6_]^2+^ (1)

## 10. Conversion of 1 into 2

**Figure 5:**
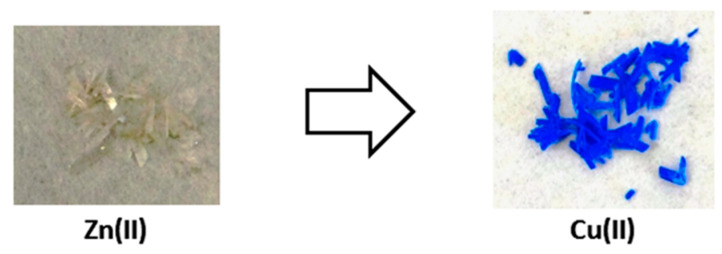
Conversion of crystals of **1** to **2**, indicating the nature of crystallinity.

**Figure 6:**
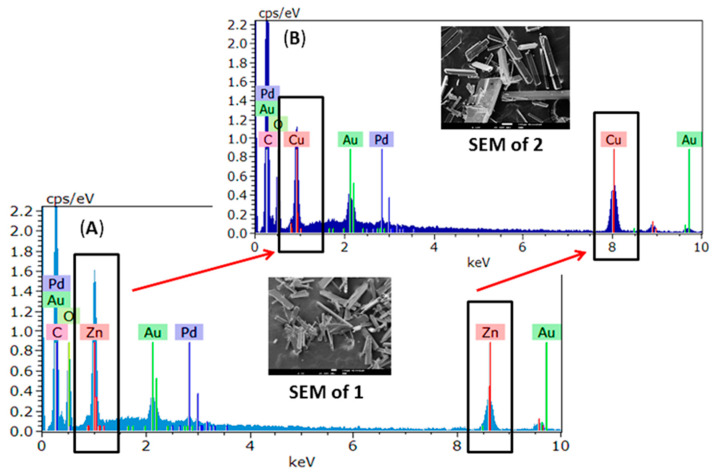
EDS of **1** (**A**) and **2** (**B**), showing the complete transformation. Inset shows SEM images of **1** and **2**, respectively.

**Figure 7:**
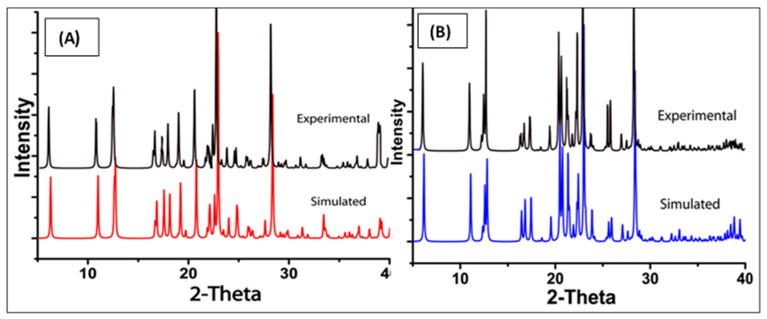
Simulated and experimental PXRD patterns of **1** (**A**) and **2** (**B**).

The conversion of **1** into **2** was evidenced by the color change from colorless to blue (Figure 5). During such conversion process, no loss in crystal quality was observed as evidenced by SEM analysis (inset of Figure 6) as well as by X-ray (see above). The complete conversion of **1** to **2** was validated by SEM–EDS method (Figure 6). Figure 7 presents the PXRD patterns of **1** and **2** both calculated from their crystal structures and experimentally obtained from the bulk materials, with undeniable resemblance.

The recurrent attempted synthesis and isolation of **2** by hydrothermal reaction between **HL** and Cu(NO_3_)_2_ × 3H_2_O were unsuccessful. Moreover, by soaking crystals of **2** in a DMF:H_2_O (1:1, *v*/*v*) solvent mixture and subsequent addition of an aqueous solution containing a molar excess of Zn(NO_3_)_2_ × 6H_2_O, no transmetalation reaction took place even after more than a month, showing the irreversibility of the process on account of the stronger coordination of the ligand to Cu(II) in comparison with Zn(II). Furthermore, to know if Zn(II) in **1** could be replaced by any other transition metal ion, apart from Cu(II), analogous procedures to that mentioned above for the **1** to **2** conversion were followed using salts of Mn^2+^, Co^2+^, Ni^2+^, Pb^2+^ and Cd^2+^, but no transmetalation was observed on the basis of EDS analyses (Figure S2), thus, disclosing a curious selectivity towards Cu(II).

## 11. Catalytic Oxidation of Styrene to Benzaldehyde

**Scheme 2:**
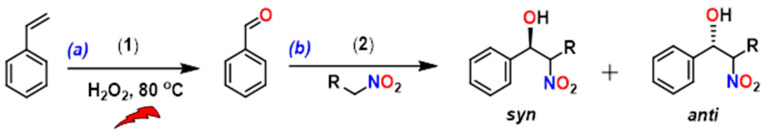
(**a**) Microwave-assisted peroxidative oxidation of styrene to benzaldehyde catalyzed by *trans*-[CuL_2_(H_2_O)_2_] (**2**); (**b**) Nitroaldol (Henry) coupling of benzaldehyde with nitroalkanes (R = H or Me) catalyzed by *cis*-[ZnL_2_(H_2_O)_2_] (**1**).

Both complexes **1** and **2** were tested for the peroxidative oxidation of styrene. Complex **1** was not active as a catalyst, since the styrene conversion obtained in its presence was similar to the one obtained in blank tests (without any metal catalyst). On the contrary, the copper complex **2** was successfully tested as a heterogeneous catalyst for the oxidation of styrene by aqueous hydrogen peroxide, in acetonitrile, yielding selectively (>99%) benzaldehyde (Scheme 2a) under optimized reaction conditions (microwave irradiation, 80 °C, 30 min., 600 rpm, 10 W, *n*_oxidant_/*n*_substrate_ = 2) up to 66% of conversion and a turnover frequency (moles of product per mole of catalyst per unit of time, TOF) of 132 h^−1^.

When a decrease in the selectivity for benzaldehyde was found (higher oxidant to styrene molar ratios, *n*_oxidant_/*n*_substrate_ ≥ 4), although being the major product, the formation of 1-phenylethane-1,2-diol (up to 23%) was detected. This can suggest that the mechanism for styrene oxidation is initiated by the Cu-assisted formation of oxygen-centered radicals (hydroxyl and peroxyl, similar to Fenton chemistry) which oxidize styrene, what is also suggested by the reaction inhibition upon addition of the radical scavenger 2,2,6,6-tetramethylpiperidine-1-oxyl (TEMPO) to the reaction mixture. Moreover, the epoxide product was not detected, neither acetophenone nor phenylacetaldehyde (from isomerization of the epoxide).

Besides, an increase in the microwave irradiation time led to the formation of benzoic acid by overoxidation of benzaldehyde. In fact, if the desired product would be benzoic acid, full conversion of styrene could be obtained performing the reaction under MW irradiation during 2 h at 80 °C with an increased amount of hydrogen peroxide. Higher temperatures than 80 °C were not tested due to safety reasons.

The recyclability of complex **2** was examined by recovering it through centrifugation (see Experimental). Although a slight color change was detected for the recovered catalyst, a new cycle was run with it by addition of new typical portions of all other reagents. Unfortunately, the catalytic activity of recovered **2** for the second cycle was significantly lower (up to 17% conversion) what prevented its reuse.

## 12. Catalytic Benzaldehyde Nitroaldol C-C Coupling

**Figure 8:**
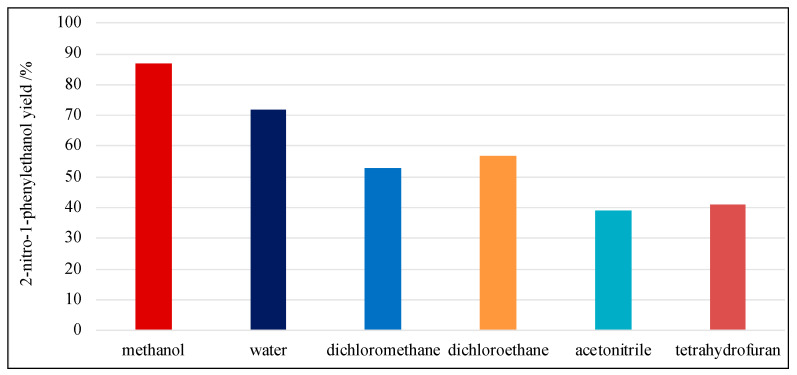
Effect of the solvent type on the yield of the product of the Henry coupling of benzaldehyde with nitromethane catalyzed by *cis*-[ZnL_2_(H_2_O)_2_] (**1**).

Complex **1** showed a good activity as a heterogeneous catalyst for the Henry coupling reaction of benzaldehyde with nitroalkanes yielding β-nitro alcohols (Scheme 2b) up to 87% conversion under optimized reaction conditions (24 h at 60 °C in MeOH, benzaldehyde: nitroalkane 1:3 and 10 mol% of **1** vs. benzaldehyde). Moreover, acceptable values of *syn*:*anti* selectivity for the diastereoisomers of 1-phenyl-2-nitropropanol, formed by the nitroaldol coupling using nitroethane, were achieved (up to 66:34 *syn*:*anti*), with superior selectivity for the *syn* isomer. The efficiency of the nitroaldol coupling was shown to be dependent on the solvent: protic solvents (e.g., water or methanol) led to higher yields of β-nitro alcohols than aprotic ones (e.g., acetonitrile or THF) (see Figure 8 for 2-nitro-1-phenylethanol). With the Cu(II) complex **2** as a catalyst, only 32 to 68% benzaldehyde conversions were achieved after 24 h at 60 °C. Following previous studies [18,48,49,50,51], the reaction mechanism should involve metal-assisted (upon coordination) deprotonation of nitroalkane to produce the nitronate species and activation of benzaldehyde for its electrophilic attack to the nitronate. Therefore, the Zn(II) complex **1** acts more efficiently as a Lewis acid for the aforementioned activation process than its related Cu(II) **2**.

Under solvent-free conditions, a significant decrease on the β-nitro alcohol production was observed (19% after 24 h at 60 °C, with benzaldehyde: nitroalkene 1:3 and 10 mol% of **1** vs. benzaldehyde). Besides, the reaction does not show any progression in the absence of **1**. The recyclability of complex **1** was impaired by its decomposition during its recovering from the reaction mixture.

## 13. Conclusions

The mononuclear Zn(II) complex *cis*-[ZnL_2_(H_2_O)_2_] (**1**) was synthesized through hydrothermal reaction using 4-(pyridin-3-ylcarbamoyl)benzoic acid (HL) as the ligand source, and can be irreversibly transmetalated to the Cu(II) analogue *trans*-[CuL_2_(H_2_O)_2_] (**2**). The thermodynamic feasibility of transmetalation is evidenced by DFT studies. The catalytic activity of **1** and **2** were tested for oxidation and for CC-coupling reactions. Compound **2** with the redox active Cu(II) site acts as heterogeneous catalyst for the microwave-assisted selective oxidation of styrene to benzaldehyde by aqueous hydrogen peroxide. In contrast, compound **1** with the strong Lewis acid Zn(II) site was found to be an active catalyst towards the nitroaldol (Henry) reaction between benzaldehyde and nitroalkanes to afford the corresponding β-nitro alcohols. Further, the reaction of benzaldehyde with nitroethane in the presence of **1** produced 2-nitro-1-phenylpropanol in the *syn* and the *anti* diastereoisomeric forms, with a considerable higher selectivity towards the *syn* isomer.

## Figures and Tables

**Table 1:** Crystal data and structure refinement details for compounds **1** and **2**.

Identification Name	1	2
Formulae	C_26_H_22_N_4_O_8_Zn	C_26_H_22_N_4_O_8_Cu
Mol. wt.	583.84	582.01
Crystal system	Monoclinic	Monoclinic
Space group	C2/c	P2_1_/c
Temperature/K	296	296
Wavelength/Å	0.71073	0.71073
*a*/Å	32.201(2)	7.9726(4)
*b*/Å	5.1266(3)	5.1600(3)
*c*/Å	16.0629(11)	28.6218(14)
β/°	119.367(2)	91.262(2)
V/Å^3^	2311.0(3)	1177.18(11)
Z	4	2
Density/Mgm^−3^	1.678	1.642
Abs. Coeff./mm^−1^	1.127	0.991
F(000)	1200	598
Refl. collected	22855	15987
R(int)	0.0658	0.0263
Refl. Obs/unique	2118/1838	2160/2118
Max. *θ*/°	25.392	25.412
Ranges (h, k, l)	−38 <= h <= 38	−9 <= h <= 9
	−6 <= k <= 6	−6 <= k <= 6
	−19 <= l <= 19	−34 <= l <= 34
Complete to *θ* (%)	99.8	99.3
N° Parameters	186	187
R1 [I > 2σ(I)]	0.0290	0.0701
wR2 [I > 2σ(I)]	0.0618	0.1588
R1 [all data]	0.0386	0.0710
wR2 [all data]	0.0649	0.1591
GOF	1.114	1.355

## Supplementary Materials

The following are available online, Figure S1. IR spectra of HL, 1 and 2, Figure S2. EDS of the samples to identify any transformation of the metal ions using Pb(II) (A) Co(II) (B), Cd(II) (C), Mn(II) (D), Ni(II) (E) salts, Table S1. Hydrogen bond interactions (Å, °) in compounds 1 and 2, Table S2. Cartesian atomic coordinates of the calculated equilibrium structures of **1** and **2**.

## References

[1] Arthurs R.A., Hughes D.L., Richards C.J. (2019). Ferrocenyloxazoline-Derived Planar Chiral Palladacycles: C-H Activation, Transmetalation, and Reversal of Diastereoselectivity. Organometallics.

[2] Zeckert K., Fuhrmann D. (2019). Bis(2-pyridyl) and Tris(2-pyridyl) Compounds of Gallium and Indium via a Redox-Transmetalation Route. Inorg. Chem..

[3] Xie J., Boyn J.N., Filatov A.S., McNeece A.J., Mazziotti D.A., Anderson J.S. (2020). Redox, transmetalation, and stacking properties of tetrathiafulvalene-2,3,6,7-tetrathiolate bridged tin, nickel, and palladium compounds. Chem. Sci..

[4] Fox O.D., Cookson J., Wilkinson E.J.S., Drew M.G.B., MacLean E.J., Teat S.J., Beer P.D. (2006). Nanosized polymetallic resorcinarene-based host assemblies that strongly bind fullerenes. J. Am. Chem. Soc..

[5] Sarma R.J., Otto S., Nitschke J.R. (2007). Disulfides, imines, and metal coordination within a single system: Interplay between three dynamic equilibria. Chem. A Eur. J..

[6] Patel U., Sharma S., Singh H.B., Dey S., Jain V.K., Wolmershäuser G., Butcher R.J. (2010). Intermetallic bonds in metallophilic mercuraazametallamacrocycles of synthetic design. Organometallics.

[7] Park Y.J., Ryu J.Y., Hwang S., Park K.H., Lee J.M., Cho S., Lee S., Saha M.L., Stang P.J., Lee J. (2017). Cationic Ti Complexes with Three [N,O]-Type Tetrazolyl Ligands: Ti Fe Transmetalation within Fe Metallascorpionate Complexes. Inorg. Chem..

[8] Baya M., Belío Ú., Campillo D., Fernández I., Fuertes S., Martín A. (2018). Pt–M Complexes (M = Ag, Au) as Models for Intermediates in Transmetalation Processes. Chem. A Eur. J..

[9] Ube H., Endo K., Sato H., Shionoya M. (2019). Synthesis of Hetero-multinuclear Metal Complexes by Site-Selective Redox Switching and Transmetalation on a Homo-multinuclear Complex. J. Am. Chem. Soc..

[10] Auth T., Koszinowski K., O’Hair R.A.J. (2020). Dissecting Transmetalation Reactions at the Molecular Level: Phenyl Transfer in Metal Borate Complexes. Organometallics.

[11] Imanishi K., Wahyudianto B., Kojima T., Yoshinari N., Konno T. (2020). A 116-Nuclear Metallosupramolecular Cage-of-Cage Showing Multistep Single-Crystal-to-Single-Crystal Transformation. Chem. A Eur. J..

[12] De D., Neogi S., Sañudo E.C., Bharadwaj P.K. (2015). Single-Crystal to Single-Crystal Linker Substitution, Linker Place Exchange, and Transmetalation Reactions in Interpenetrated Pillared-Bilayer Zinc(II) Metal-Organic Frameworks. Chem. A Eur. J..

[13] Balestri D., Bassanetti I., Canossa S., Gazzurelli C., Bacchi A., Bracco S., Comotti A., Pelagatti P. (2018). Changing the Dress to a MOF through Fluorination and Transmetalation. Structural and Gas-Sorption Effects. Cryst. Growth Des..

[14] Bommakanti S., Venkataramudu U., Das S.K. (2019). Functional Coordination Polymers from a Bifunctional Ligand: A Quantitative Transmetalation via Single Crystal to Single Crystal Transformation. Cryst. Growth Des..

[15] Kassie A.A., Duan P., McClure E.T., Schmidt-Rohr K., Woodward P.M., Wade C.R. (2019). Postsynthetic Metal Exchange in a Metal-Organic Framework Assembled from Co(III) Diphosphine Pincer Complexes. Inorg. Chem..

[16] Xu W.Y., Tian X.Z., Feng X.F., Huang H.X., Sun G.M., Song Y.M., Luo F. (2012). Three new acylamide ligands formed in situ and their application in constructing metal-organic frameworks. CrystEngComm.

[17] Karmakar A., Guedes Da Silva M.F.C., Pombeiro A.J.L. (2014). Zinc metal-organic frameworks: Efficient catalysts for the diastereoselective Henry reaction and transesterification. Dalt. Trans..

[18] Karmakar A., Martins L.M.D.R.S., Hazra S., Guedes Da Silva M.F.C., Pombeiro A.J.L. (2016). Metal-Organic Frameworks with Pyridyl-Based Isophthalic Acid and Their Catalytic Applications in Microwave Assisted Peroxidative Oxidation of Alcohols and Henry Reaction. Cryst. Growth Des..

[19] Karmakar A., Pombeiro A.J.L. (2019). Recent advances in amide functionalized metal organic frameworks for heterogeneous catalytic applications. Coord. Chem. Rev..

[20] Karmakar A., Paul A., Rúbio G.M.D.M., Soliman M.M.A., Guedes da Silva M.F.C., Pombeiro A.J.L. (2019). Highly Efficient Bifunctional Amide Functionalized Zn and Cd Metal Organic Frameworks for One-Pot Cascade Deacetalization–Knoevenagel Reactions. Front. Chem..

[21] Paul A., Martins L.M.D.R.S., Karmakar A., Kuznetsov M.L., Novikov A.S., Guedes da Silva M.F.C., Pombeiro A.J.L. (2020). Environmentally benign benzyl alcohol oxidation and C-C coupling catalysed by amide functionalized 3D Co(II) and Zn(II) metal organic frameworks. J. Catal..

[22] Ma Z., Wei L., Alegria E.C.B.A., Martins L.M.D.R.S., Guedes Da Silva M.F.C., Pombeiro A.J.L. (2014). Synthesis and characterization of copper(ii) 4′-phenyl-terpyridine compounds and catalytic application for aerobic oxidation of benzylic alcohols. Dalt. Trans..

[23] Timokhin I., Pettinari C., Marchetti F., Pettinari R., Condello F., Galli S., Alegria E.C.B.A., Martins L.M.D.R.S., Pombeiro A.J.L. (2015). Novel coordination polymers with (Pyrazolato)-based tectons: Catalytic activity in the peroxidative oxidation of alcohols and cyclohexane. Cryst. Growth Des..

[24] Hazra S., Martins L.M.D.R.S., Guedes da Silva M.F.C., Pombeiro A.J.L. (2017). Sulfonated Schiff base dimeric and polymeric copper(II) complexes: Temperature dependent synthesis, crystal structure and catalytic alcohol oxidation studies. Inorg. Chim. Acta.

[25] Ribeiro A.P.C., Martins L.M.D.R.S., Carabineiro S.A.C., Buijnsters J.G., Figueiredo J.L., Pombeiro A.J.L. (2018). Heterogenized C-Scorpionate Iron(II) Complex on Nanostructured Carbon Materials as Recyclable Catalysts for Microwave-Assisted Oxidation Reactions. ChemCatChem.

[26] Friedrich E., Wright B. (2012). Benzaldehyde—Ullmann’s. Encyclopedia of Industrial Chemistry.

[27] Marotta R., Di Somma I., Spasiano D., Andreozzi R., Caprio V. (2011). Selective oxidation of benzyl alcohol to benzaldehyde in water by TiO_2_/Cu(II)/UV solar system. Chem. Eng. J..

[28] Forsyth A.C., Paton R.M., Watt I. (1989). Highly selective base-catalysed additions of nitromethane to levoglucosenone. Tetrahedron Lett..

[29] Fitch W.W. (1994). Fitch. Tetrahedron Lett..

[30] Cwik A., Fuchs A., Hell Z., Clacens J.M. (2005). Nitroaldol-reaction of aldehydes in the presence of non-activated Mg:Al 2:1 hydrotalcite; A possible new mechanism for the formation of 2-aryl-1,3-dinitropropanes. Tetrahedron.

[31] Mayani V.J., Abdi S.H.R., Kureshy R.I., Khan N.U.H., Das A., Bajaj H.C. (2010). Heterogeneous chiral copper complexes of amino alcohol for asymmetric nitroaldol reaction. J. Org. Chem..

[32] Kopylovich M.N., Mac Leod T.C.O., Mahmudov K.T., Guedes Da Silva M.F.C., Pombeiro A.J.L. (2011). Zinc(ii) ortho-hydroxyphenylhydrazo-β-diketonate complexes and their catalytic ability towards diastereoselective nitroaldol (Henry) reaction. Dalt. Trans..

[33] Kopylovich M.N., Mizar A., Guedes Da Silva M.F.C., Mac Leod T.C.O., Mahmudov K.T., Pombeiro A.J.L. (2013). Template syntheses of copper(II) complexes from arylhydrazones of malononitrile and their catalytic activity towards alcohol oxidations and the nitroaldol reaction: Hydrogen bond-assisted ligand liberation and E/Z isomerisation. Chem. A Eur. J..

[34] Mahmudov K.T., Kopylovich M.N., Haukka M., Mahmudova G.S., Esmaeila E.F., Chyragov F.M., Pombeiro A.J.L. (2013). Aqua complex of iron(III) and 5-chloro-3-(2-(4,4-dimethyl-2,6- dioxocyclohexylidene) hydrazinyl)-2-hydroxybenzenesulfonate: Structure and catalytic activity in Henry reaction. J. Mol. Struct..

[35] Karmakar A., Hazra S., Guedes Da Silva M.F.C., Pombeiro A.J.L. (2014). Synthesis, structure and catalytic applications of amidoterephthalate copper complexes in the diastereoselective Henry reaction in aqueous medium. New J. Chem..

[36] Paul A., Karmakar A., Fátima M., Guedes Da Silva C., Pombeiro A.J.L. (2015). Amide Functionalized Metal Organic Frameworks for Diastereoselective Nitroaldol (Henry) Reaction in Aqueous Medium. RSC Adv..

[37] Jin F., Yang X.F., Li S.L., Zheng Z., Yu Z.P., Kong L., Hao F.Y., Yang J.X., Wu J.Y., Tian Y.P. (2012). Role of anions in preparing silver(i) complexes with a new multidentate ligand: Polymorphs, structures and nonlinear optical properties. CrystEngComm.

[38] Sheldrick G.M. SADABS.

[39] Altomare A., Burla M.C., Camalli M., Cascarano G.L., Giacovazzo C., Guagliardi A., Moliterni A.G.G., Polidori G., Spagna R. (1999). SIR97: A new tool for crystal structure determination and refinement. J. Appl. Crystallogr..

[40] Sheldrick G.M. (2008). A short history of SHELX. Acta Crystallogr. Sect. A Found. Crystallogr..

[41] Farrugia L.J. (2012). WinGX and ORTEP for Windows: An update. J. Appl. Crystallogr..

[42] Spek A.L. (1990). PLATON, An integrated tool for the analysis of the results of a single crystal structure determination. Acta Crystallogr. Sect. A.

[43] Zhao Y., Truhlar D.G. (2008). The M06 suite of density functionals for main group thermochemistry, thermochemical kinetics, noncovalent interactions, excited states, and transition elements: Two new functionals and systematic testing of four M06-class functionals and 12 other function. Theor. Chem. Acc..

[44] Frisch M.J., Trucks G.W., Schlegel H.B., Scuseria G.E., Robb M.A., Cheeseman J.R., Scalmani G., Barone V., Mennucci B., Petersson G.A. (2013). Gaussian 09, Revis. D.01.

[45] Marenich A.V., Cramer C.J., Truhlar D.G. (2009). Universal solvation model based on solute electron density and on a continuum model of the solvent defined by the bulk dielectric constant and atomic surface tensions. J. Phys. Chem. B.

[46] Denmark S.E., Kesler B.S., Moon Y.C. (1992). Inter-and Intramolecular [4 + 2] Cycloadditions of Nitroalkenes with Olefins. 2-Nitrostyrenes. J. Org. Chem..

[47] Bulbule V.J., Deshpande V.H., Velu S., Sudalai A., Sivasankar S., Sathe V.T. (1999). Heterogeneous henry reaction of aldehydes: Diastereoselective synthesis of nitroalcohol derivatives over Mg-Al hydrotalcites. Tetrahedron.

[48] Qi N., Liao R.-Z., Yu J.-G., Liu R.-Z. (2009). DFT study of the asymmetric nitroaldol (Henry) reaction catalyzed by a dinuclear Zn complex. J. Comput. Chem..

[49] Pettinari C., Marchetti F., Cerquetella A., Pettinari R., Monari M., Mac Leod T.C.O., Martins L.M.D.R.S., Pombeiro A.J.L. (2011). Coordination chemistry of the (η6-p-cymene)ruthenium(II) fragment with bis-, tris-, and tetrakis(pyrazol-1-yl)borate ligands: Synthesis, structural, electrochemical, and catalytic diastereoselective nitroaldol reaction studies. Organometallics.

[50] Martins L.M.D.R.S. (2019). C-scorpionate complexes: Ever young catalytic tools. Coord. Chem. Rev..

[51] Rocha B.G.M., Mac Leod T.C.O., Guedes da Silva M.F.C., Luzyanin K.V., Martins L.M.D.R.S., Pombeiro A.J.L. (2014). NiII, CuII and ZnII complexes with a sterically hindered scorpionate ligand (TpmsPh) and catalytic application in the diasteroselective nitroaldol (Henry) reaction. Dalt. Trans..

