# Supplementary material for: Zn(II)-to-Cu(II) Transmetalation in an Amide Functionalized Complex and Catalytic Applications in Styrene Oxidation and Nitroaldol Coupling
Source: Molecules. 2020 Jun 6;25(11):2644. doi: 10.3390/molecules25112644 (PMC7321079; doi:10.3390/molecules25112644)
Supplement: Supplementary file 1 [file molecules-25-02644-s001.pdf]

*molecules*

*Supplementary Materials*

# Zn(II)-to-Cu(II) Transmetalation in an Amide Functionalized Complex and Catalytic Applications in Styrene Oxidation and Nitroaldol Coupling

**Anup Paul *, Luísa M. D. R. S. Martins *, Anirban Karmakar, Maxim L. Kuznetsov, M. Fátima C. Guedes da Silva * and Armando J. L. Pombeiro ***

Centro de Química Estrutural, Instituto Superior Técnico, Universidade de Lisboa, 1049-001 Lisboa, Portugal

* Correspondence: anuppaul@tecnico.ulisboa.pt (A.P.), luisammartins@tecnico.ulisboa.pt (L.M.D.R.S.M.), fatima.guedes@tecnico.ulisboa.pt (M.F.C.G.d.S.), pombeiro@tecnico.ulisboa.pt (A.J.L.P)

**Contents:**

**Figure S1.** IR spectra of **HL**, **1** and **2**.
**Figure S2.** EDS of the samples to identify any transformation of the metal ions using Pb(II) (A) Co(II) (B), Cd(II) (C), Mn(II) (D), Ni(II) (E) salts.
**Table S1.** Hydrogen bond interactions (Å, °) in compounds **1** and **2**.
**Table S2.** Cartesian atomic coordinates of the calculated equilibrium structures of **1** and **2**.

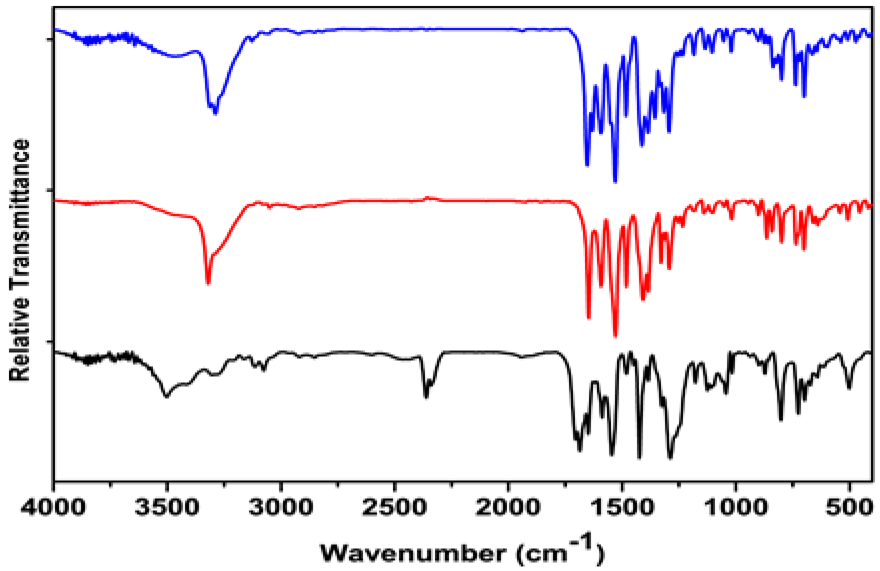


**Figure S1.** IR spectra of **HL** (black), **1** (red) and **2** (blue).

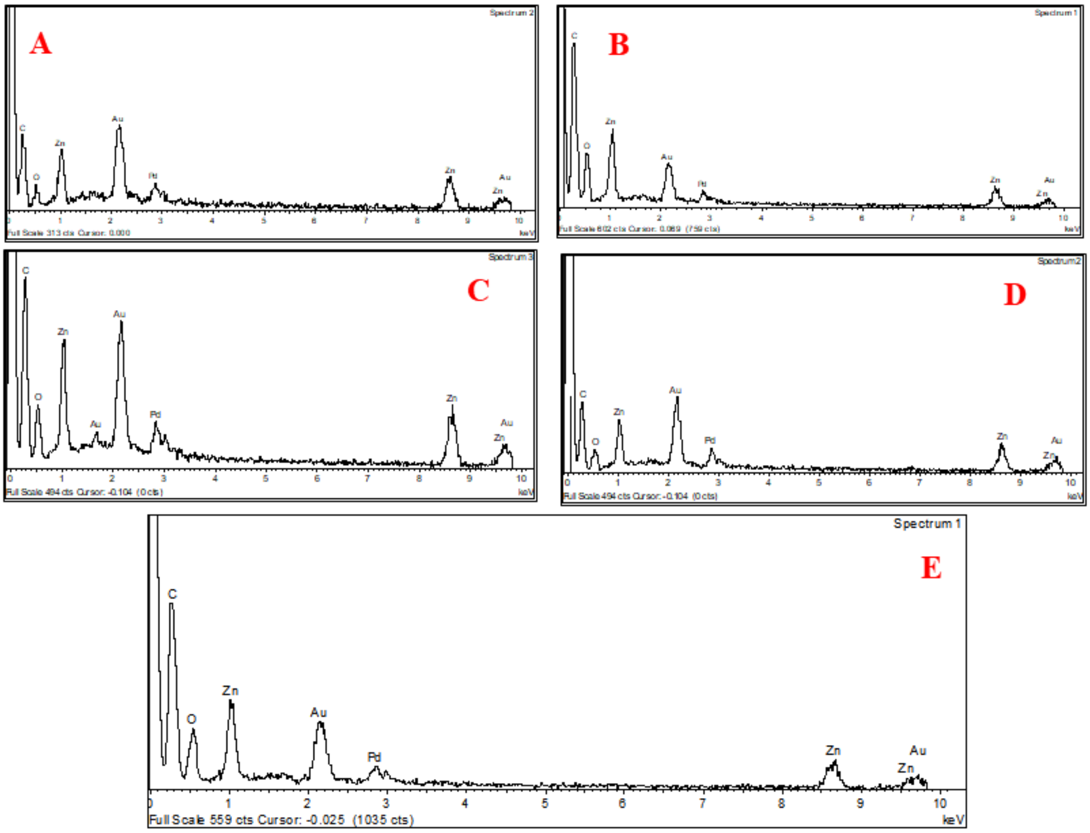


**Figure S2.** EDS of the samples to identify any eventual replacement of Zn(II) in **1** by another metal ion, using Pb(II) (A) Co(II) (B), Cd(II) (C), Mn(II) (D) or Ni(II) (E) salts.

**Table S1.** Hydrogen bond geometry (Å, °) in compounds 1 and 2.

| Compound | D-H···A | D···H (Å) | H···A (Å) | D···A (Å) | <D–H···A(°) |
|---|---|---|---|---|---|
| **1** | N1-H1N···O3 | 0.78(3) | 2.240(3) | 3.006(2) | 168(3) |
| | O4-H4B···N2 | 0.878(16) | 1.857(17) | 2.721(2) | 168(2) |
| | O4-H4A···O2 | 0.855(17) | 1.876(18) | 2.714(2) | 166(2) |
| **2** | N1-H1N···O3 | 0.88(2) | 2.17(3) | 3.013(6) | 158(6) |
| | O4-H4A···O2 | 0.78(7) | 1.90(8) | 2.667(6) | 168(7) |
| | O4-H4B···N2 | 0.95(7) | 1.86(7) | 2.729(6) | 151(6) |

**Table S2.** Cartesian atomic coordinates (in Å) of the calculated equilibrium structures (nuclear charges are given in the first column).

| | $[Zn(H_2O)_6]^{2+}$ | | |
|---|---|---|---|
| 30 | −0.000987 | −0.000453 | 0.000563 |
| 8 | 1.373575 | 0.193074 | −1.534027 |
| 8 | −1.375843 | −0.195658 | 1.534704 |
| 8 | −1.537983 | 0.312787 | −1.349393 |
| 8 | 1.537129 | −0.312705 | 1.349421 |
| 8 | 0.082826 | 2.030045 | 0.403932 |
| 8 | −0.086330 | −2.030555 | −0.403645 |
| 1 | 1.170556 | 0.408141 | −2.458538 |
| 1 | 2.340630 | 0.174385 | −1.451950 |
| 1 | −2.342851 | −0.175432 | 1.452429 |
| 1 | −1.173400 | −0.409973 | 2.459516 |
| 1 | −1.994582 | −0.372578 | −1.863266 |
| 1 | −1.952535 | 1.161027 | −1.574630 |
| 1 | 1.952549 | −1.160557 | 1.574537 |
| 1 | 1.991640 | 0.372742 | 1.865033 |
| 1 | −0.435262 | 2.484859 | 1.087320 |
| 1 | 0.602006 | 2.708032 | −0.057363 |
| 1 | −0.603606 | −2.708843 | 0.059324 |
| 1 | 0.431578 | −2.485232 | −1.087256 |

| | $[Cu(H_2O)_6]^{2+}$ | | |
|---|---|---|---|
| 29 | −0.015578 | 0.006754 | 0.007245 |
| 8 | 0.680907 | 1.927917 | 0.342005 |
| 8 | −0.600413 | −1.941678 | −0.371350 |
| 8 | −1.975058 | 0.721490 | 0.542832 |
| 8 | 1.982761 | −0.686501 | −0.507286 |
| 8 | 0.431531 | −0.533421 | 1.823275 |
| 8 | −0.452098 | 0.541907 | −1.810898 |
| 1 | 0.132464 | 2.669867 | 0.645065 |
| 1 | 1.602307 | 2.234004 | 0.341672 |
| 1 | −1.485812 | −2.340164 | −0.393270 |
| 1 | 0.028583 | −2.620763 | −0.664808 |
| 1 | −2.595379 | 1.028481 | −0.137760 |
| 1 | −2.488309 | 0.669479 | 1.364729 |
| 1 | 2.507618 | −0.649608 | −1.322604 |
| 1 | 2.592956 | −0.985692 | 0.185599 |
| 1 | 0.097414 | −1.364731 | 2.201340 |
| 1 | 0.581268 | 0.092178 | 2.552033 |
| 1 | −0.609066 | −0.085589 | −2.536394 |
| 1 | −0.109499 | 1.368471 | −2.191611 |

| | 1 | | |
|---|---|---|---|
| 30 | 0.000000 | 0.000000 | 0.100930 |
| 8 | 1.602261 | 0.072160 | 1.365498 |

| | | | |
|---|---|---|---|
| 8 | −0.117239 | 2.240313 | 1.651051 |
| 8 | −0.074746 | 1.783560 | −0.540223 |
| 8 | −0.798632 | 8.531380 | −2.007689 |
| 7 | −0.024570 | 9.110681 | 0.064662 |
| 7 | 0.565098 | 12.539986 | 1.138478 |
| 6 | −0.126740 | 2.600482 | 0.452539 |
| 6 | −0.202350 | 4.047832 | 0.113054 |
| 6 | 0.000000 | 10.509477 | −0.028588 |
| 6 | −0.343491 | 12.624386 | −1.073234 |
| 1 | −0.664010 | 13.229759 | −1.918477 |
| 6 | −0.427505 | 11.240011 | −1.138613 |
| 1 | −0.804561 | 10.726304 | −2.015784 |
| 6 | 0.154835 | 13.231037 | 0.073103 |
| 1 | 0.229517 | 14.316876 | 0.142365 |
| 6 | 0.483928 | 11.219393 | 1.076700 |
| 1 | 0.822809 | 10.667400 | 1.960573 |
| 6 | −0.339544 | 6.771382 | −0.483117 |
| 6 | −0.183482 | 4.476358 | −1.215611 |
| 1 | −0.110582 | 3.734180 | −2.006843 |
| 6 | −0.257707 | 5.828202 | −1.509705 |
| 1 | −0.257574 | 6.184659 | −2.537286 |
| 6 | −0.420443 | 8.208047 | −0.894515 |
| 6 | −0.305768 | 4.985326 | 1.140500 |
| 1 | −0.339491 | 4.633198 | 2.168592 |
| 6 | −0.375083 | 6.338448 | 0.845467 |
| 1 | −0.500575 | 7.049617 | 1.661726 |
| 1 | 0.384867 | 8.732298 | 0.910058 |
| 1 | 1.435808 | −0.732636 | 1.896175 |
| 1 | 1.366585 | 0.851682 | 1.905988 |
| 8 | −1.602261 | −0.072160 | 1.365498 |
| 8 | 0.117239 | −2.240313 | 1.651051 |
| 8 | 0.074746 | −1.783560 | −0.540223 |
| 8 | 0.798632 | −8.531380 | −2.007689 |
| 7 | 0.024570 | −9.110681 | 0.064662 |
| 7 | −0.565098 | −12.539986 | 1.138478 |
| 6 | 0.126740 | −2.600482 | 0.452539 |
| 6 | 0.202350 | −4.047832 | 0.113054 |
| 6 | 0.000000 | −10.509477 | −0.028588 |
| 6 | 0.343491 | −12.624386 | −1.073234 |
| 1 | 0.664010 | −13.229759 | −1.918477 |
| 6 | 0.427505 | −11.240011 | −1.138613 |
| 1 | 0.804561 | −10.726304 | −2.015784 |
| 6 | −0.154835 | −13.231037 | 0.073103 |
| 1 | −0.229517 | −14.316876 | 0.142365 |
| 6 | −0.483928 | −11.219393 | 1.076700 |
| 1 | −0.822809 | −10.667400 | 1.960573 |
| 6 | 0.339544 | −6.771382 | −0.483117 |
| 6 | 0.183482 | −4.476358 | −1.215611 |
| 1 | 0.110582 | −3.734180 | −2.006843 |
| 6 | 0.257707 | −5.828202 | −1.509705 |
| 1 | 0.257574 | −6.184659 | −2.537286 |
| 6 | 0.420443 | −8.208047 | −0.894515 |
| 6 | 0.305768 | −4.985326 | 1.140500 |
| 1 | 0.339491 | −4.633198 | 2.168592 |
| 6 | 0.375083 | −6.338448 | 0.845467 |
| 1 | 0.500575 | −7.049617 | 1.661726 |

| | | | |
|---|---|---|---|
| 1 | −0.384867 | −8.732298 | 0.910058 |
| 1 | −1.435808 | 0.732636 | 1.896175 |
| 1 | −1.366585 | −0.851682 | 1.905988 |
| trans−**2** | | | |
| 29 | −0.018271 | −1.436395 | −0.283459 |
| 8 | −0.249396 | −2.311967 | 1.446945 |
| 8 | −1.868966 | −1.198066 | −0.407453 |
| 8 | −8.923826 | 0.065012 | −1.662938 |
| 8 | −2.086819 | −0.877328 | −2.626091 |
| 7 | −8.509974 | 0.930709 | 0.413979 |
| 7 | −11.042977 | 2.479145 | 2.522776 |
| 6 | −9.790498 | 1.347445 | 0.803474 |
| 6 | −3.985148 | −0.607144 | −1.248222 |
| 6 | −6.685444 | −0.001816 | −0.873989 |
| 6 | −2.540449 | −0.913325 | −1.469736 |
| 6 | −12.153760 | 2.258904 | 1.817139 |
| 1 | −13.083242 | 2.635963 | 2.245562 |
| 6 | −9.902274 | 2.031126 | 2.020106 |
| 1 | −9.000061 | 2.221328 | 2.612148 |
| 6 | −5.854291 | −0.226228 | 0.226895 |
| 1 | −6.255371 | −0.207401 | 1.240214 |
| 6 | −6.159359 | −0.108592 | −2.163172 |
| 1 | −6.828191 | 0.041041 | −3.007657 |
| 6 | −4.817189 | −0.395817 | −2.349200 |
| 1 | −4.389096 | −0.465953 | −3.346514 |
| 6 | −12.153261 | 1.589881 | 0.599152 |
| 1 | −13.085079 | 1.434093 | 0.059589 |
| 6 | −10.957499 | 1.120587 | 0.072195 |
| 1 | −10.912242 | 0.599101 | −0.877157 |
| 6 | −8.142083 | 0.319100 | −0.762592 |
| 6 | −4.513558 | −0.530771 | 0.040043 |
| 1 | −3.862325 | −0.716515 | 0.890229 |
| 1 | −7.761423 | 1.216927 | 1.033154 |
| 1 | −0.921569 | −3.004219 | 1.494361 |
| 1 | 0.666101 | −2.679622 | 1.633754 |
| 8 | 0.310570 | −1.619351 | −2.187337 |
| 8 | 1.772544 | −0.964441 | 0.002489 |
| 8 | 8.735506 | −0.090671 | 1.986238 |
| 8 | 2.289185 | −2.633140 | 1.418847 |
| 7 | 8.537427 | 0.936490 | −0.048539 |
| 7 | 11.245822 | 2.714276 | −1.712286 |
| 6 | 9.814645 | 1.491808 | −0.207937 |
| 6 | 3.992641 | −1.128558 | 0.781396 |
| 6 | 6.633397 | −0.224461 | 0.890980 |
| 6 | 2.587365 | −1.632555 | 0.747124 |
| 6 | 12.201707 | 2.680978 | −0.781775 |
| 1 | 13.144959 | 3.164301 | −1.039002 |
| 6 | 10.091327 | 2.133248 | −1.421327 |
| 1 | 9.316757 | 2.171774 | −2.195372 |
| 6 | 5.682051 | 0.466661 | 0.135771 |
| 1 | 5.945407 | 1.389481 | −0.381138 |
| 6 | 6.246202 | −1.357422 | 1.609587 |
| 1 | 6.993534 | −1.866292 | 2.214409 |
| 6 | 4.938797 | −1.810113 | 1.549055 |
| 1 | 4.623250 | −2.694253 | 2.097832 |
| 6 | 12.029839 | 2.071299 | 0.454845 |

| | | | |
|---|---|---|---|
| 1 | 12.839008 | 2.069621 | 1.182026 |
| 6 | 10.819990 | 1.462160 | 0.759772 |
| 1 | 10.646336 | 0.972494 | 1.711359 |
| 6 | 8.060365 | 0.205469 | 1.015471 |
| 6 | 4.370518 | 0.016964 | 0.081610 |
| 1 | 3.621814 | 0.559124 | −0.490438 |
| 1 | 7.934962 | 1.009276 | −0.859197 |
| 1 | 1.061061 | −1.105545 | −2.516404 |
| 1 | −0.550639 | −1.310658 | −2.619166 |
| | cis−**2** | | |
| 29 | −0.113003 | −4.622158 | 0.100915 |
| 8 | −1.269456 | −5.945552 | 0.931580 |
| 8 | 1.241054 | −3.319397 | 0.041691 |
| 8 | 6.500552 | 1.488393 | −1.470273 |
| 8 | 2.789603 | −4.385796 | −1.196107 |
| 7 | 5.418823 | 2.296867 | 0.375461 |
| 7 | 6.273373 | 5.427938 | 2.025877 |
| 6 | 6.080555 | 3.516786 | 0.572040 |
| 6 | 3.182398 | −2.142667 | −0.570376 |
| 6 | 4.748058 | 0.168591 | −0.562442 |
| 6 | 2.356876 | −3.386263 | −0.589066 |
| 6 | 7.187747 | 5.930478 | 1.194022 |
| 1 | 7.613444 | 6.896323 | 1.468609 |
| 6 | 5.743097 | 4.255314 | 1.712331 |
| 1 | 4.994263 | 3.857151 | 2.406231 |
| 6 | 3.471194 | 0.179672 | 0.006211 |
| 1 | 3.053680 | 1.098993 | 0.416944 |
| 6 | 5.224728 | −1.002692 | −1.154805 |
| 1 | 6.209798 | −0.984655 | −1.615651 |
| 6 | 4.451119 | −2.151479 | −1.152921 |
| 1 | 4.811976 | −3.073057 | −1.603306 |
| 6 | 7.593221 | 5.278883 | 0.035687 |
| 1 | 8.343036 | 5.728184 | −0.612018 |
| 6 | 7.037874 | 4.049452 | −0.293246 |
| 1 | 7.329030 | 3.503051 | −1.184033 |
| 6 | 5.639945 | 1.367549 | −0.615059 |
| 6 | 2.693214 | −0.968947 | 0.002136 |
| 1 | 1.694056 | −0.968036 | 0.430253 |
| 1 | 4.775093 | 2.035093 | 1.112170 |
| 1 | −0.879585 | −6.473370 | 1.641329 |
| 1 | −2.039210 | −5.382792 | 1.298513 |
| 8 | 1.043540 | −6.063540 | −0.498424 |
| 8 | −1.463534 | −3.323234 | −0.029853 |
| 8 | −7.022844 | 1.369178 | 0.204375 |
| 8 | −2.935445 | −4.130383 | 1.469717 |
| 7 | −5.170359 | 2.630922 | −0.254809 |
| 7 | −5.235797 | 6.244367 | −0.705989 |
| 6 | −5.732900 | 3.907139 | −0.395964 |
| 6 | −3.366917 | −2.035559 | 0.468955 |
| 6 | −4.924864 | 0.257839 | 0.149178 |
| 6 | −2.540618 | −3.262726 | 0.666227 |
| 6 | −6.546892 | 6.493150 | −0.702842 |
| 1 | −6.842140 | 7.535894 | −0.825353 |
| 6 | −4.853763 | 4.984911 | −0.556450 |
| 1 | −3.774049 | 4.798385 | −0.561276 |
| 6 | −3.695841 | 0.143500 | −0.506714 |

| | | | |
|---|---|---|---|
| 1 | −3.350285 | 0.927758 | −1.180375 |
| 6 | −5.378927 | −0.796249 | 0.944870 |
| 1 | −6.350212 | −0.699791 | 1.425066 |
| 6 | −4.600901 | −1.929515 | 1.112659 |
| 1 | −4.931815 | −2.752426 | 1.741801 |
| 6 | −7.503092 | 5.496292 | −0.553078 |
| 1 | −8.561651 | 5.747490 | −0.557027 |
| 6 | −7.102402 | 4.176171 | −0.396529 |
| 1 | −7.816244 | 3.370389 | −0.269812 |
| 6 | −5.818554 | 1.451154 | 0.033403 |
| 6 | −2.922616 | −0.997971 | −0.350281 |
| 1 | −1.968371 | −1.102761 | −0.861197 |
| 1 | −4.158045 | 2.609557 | −0.242286 |
| 1 | 1.848057 | −5.571538 | −0.895116 |
| 1 | 0.675797 | −6.672154 | −1.153089 |
